# Supplementary material for: The Arabidopsis MYB96 Transcription Factor Mediates ABA-Dependent Triacylglycerol Accumulation in Vegetative Tissues under Drought Stress Conditions
Source: Plants (Basel). 2019 Aug 22;8(9):296. doi: 10.3390/plants8090296 (PMC6784083; doi:10.3390/plants8090296)
Supplement: Supplementary file 1 [file plants-08-00296-s001.pdf]

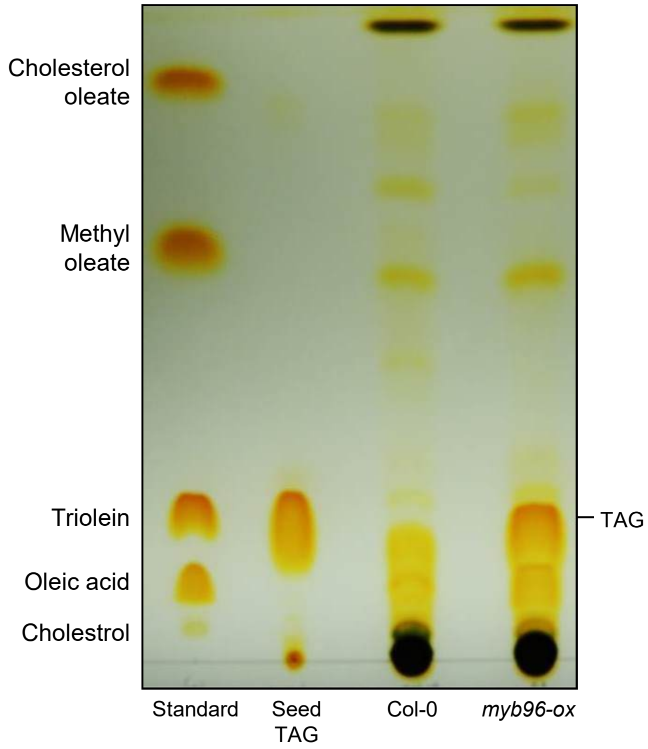

Fig. S1. TAG accumulation in *myb96-ox* seedlings.
Ten-day-old seedlings grown under long-day (LD) conditions were used to extract total lipids. Extracted lipids were separated in TLC plates. Neutral lipid standard and TAG from wild-type seeds were loaded on the left of the plate to indicate positions of the lipids.

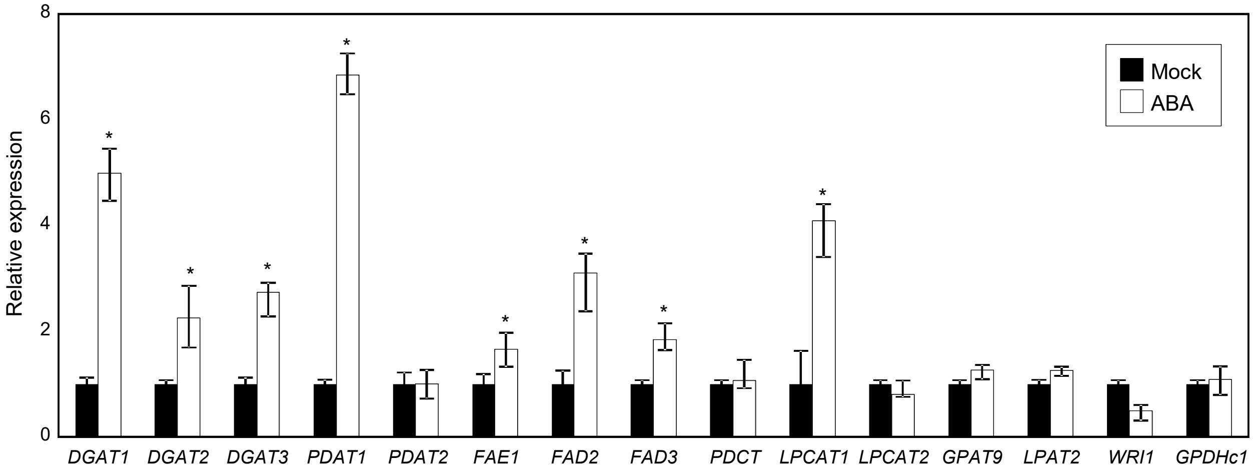

Fig. S2. Effects of ABA on transcript accumulation of lipid metabolic genes.
Ten-day-old seedlings grown under LD conditions were transferred to MS-liquid medium supplemented with 20 μM abscisic acid (ABA) and incubated for 24 h. Transcript accumulation was analyzed by quantitative real-time RT-PCR (RT-qPCR). The *eIF4a* gene was used as an internal control. Biological triplicates were averaged. Statistically significant differences between mock and A BA-treated samples are indicated by asterisks (**P*<0.05, Student's *t*-test). Bars indicate the standard error of the mean.

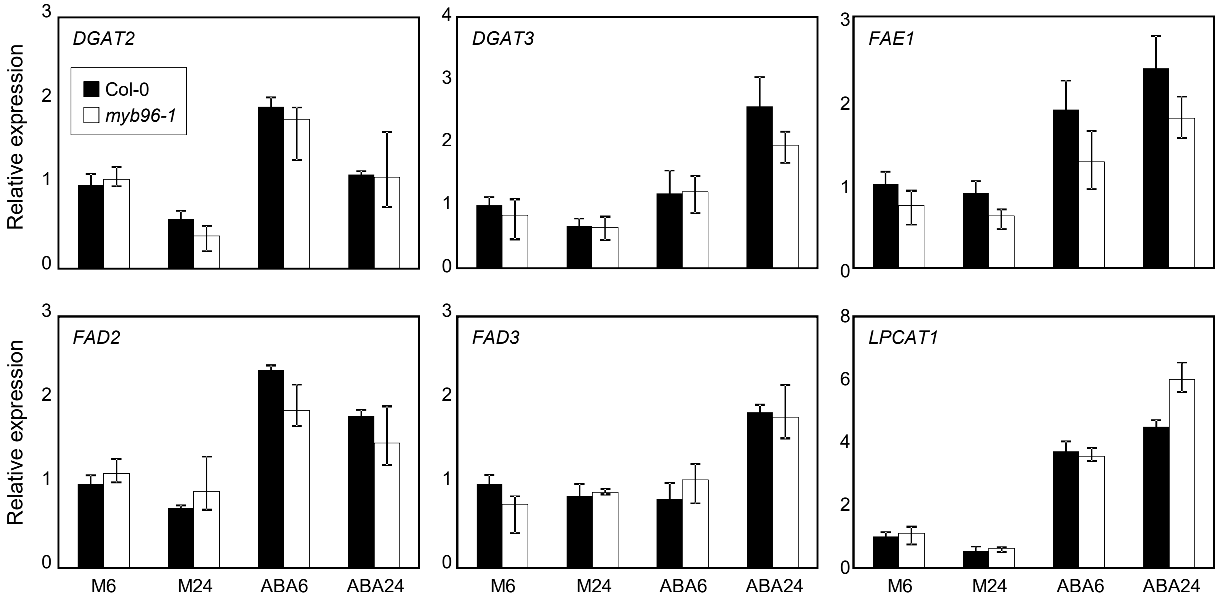

Fig. S3. Effects of ABA on transcript accumulation of lipid metabolic genes in *myb96-1*.
Ten-day-old seedlings grown under LD conditions were transferred to MS-liquid medium supplemented with 20 μM ABA and incubated for up to 24 h. Transcript accumulation was analyzed by RT-qPCR. The *eIF4a* gene was used as an internal control. Biological triplicates were averaged. Bars indicate the standard error of the mean. M, mock.

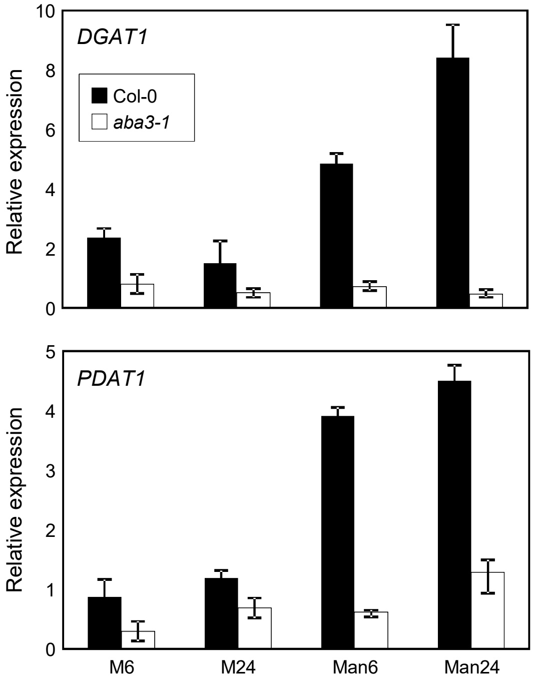

Fig. S4. Effects of osmotic stress on transcript accumulation of *DGAT1* and *PDAT1* in *aba3-1* mutant.

Ten-day-old seedlings grown under LD conditions were transferred to MS-liquid medium supplemented with 150 mM mannitol (Man) and incubated for indicated time period (h). Transcript accumulation was analyzed by RT-qPCR. The *eIF4a* gene was used as an internal control. Biological triplicates were averaged. Bars indicate the standard error of the mean. M, mock.

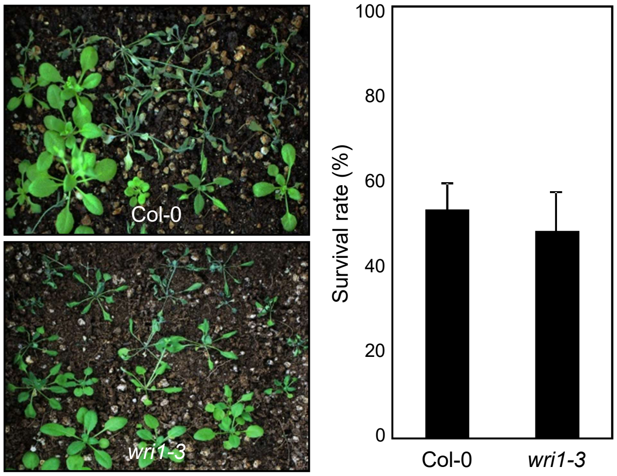

Fig. S5. Drought tolerance of *wri1-3* mutant plants.

Two-week-old plants were subjected to drought conditions by withholding water for two weeks. At least, five containers of two genotypes (30 plants/container) were evaluated in three independent experiments. Plant survival rate was determined 3 days after rewatering. Biological triplicates were averaged. Bars indicate the standard error of the mean.

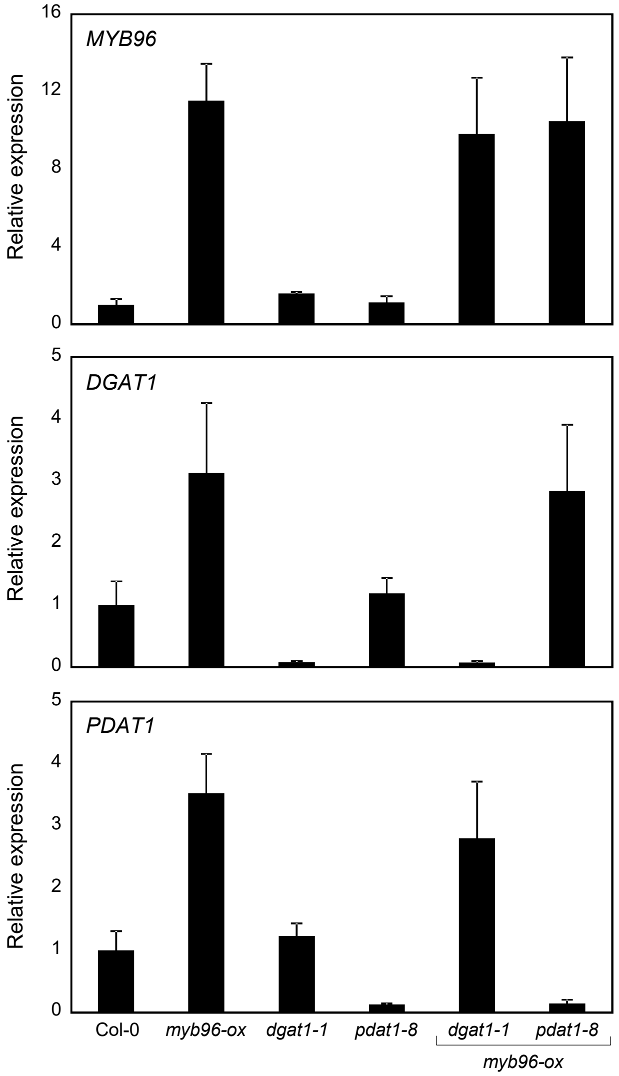

Fig. S6. Transcript accumulation of *DGAT1* and *PDAT1* in TAG-deficient mutants.
Ten-day-old seedlings grown under LD conditions were used to analyze transcript accumulation. The *eIF4a* gene was used as an internal control. Biological triplicates were averaged. Bars indicate the standard error of the mean.

Supplemental Table

Primer	Usage	Sequence
eIF4a-F	RT-qPCR	5'-TGACCACACAGTCTCTGCAA
eIF4a-R	RT-qPCR	5'-ACCAGGGAGACTTGTTGGAC
DGAT1-F	RT-qPCR	5'-TTGGATTCTGCTGGCGTTAC
DGAT1-R	RT-qPCR	5'-GCCTCTTCCACCACCGTTAT
DGAT2-F	RT-qPCR	5'-TGCGCATAGCCATGGAACAG
DGAT2-R	RT-qPCR	5'-TGGTTTACCAACGACCACATGC
DGAT3-F	RT-qPCR	5'-TGGCCAATCCTGGACAGACA
DGAT3-R	RT-qPCR	5'-AACGTTTGGGCCATCACGAC
PDAT1-F	RT-qPCR	5'-TTTCGAGGTGCTGTCAAAGG
PDAT1-R	RT-qPCR	5'-CGCCATCATCTTAGGAGCAA
PDAT2-F	RT-qPCR	5'-CCAAATTACCGGAGGCACCA
PDAT2-R	RT-qPCR	5'-TTCCTCTCCATCCCTTTGCG
FAE1-F	RT-qPCR	5'-GGAAGACTTTTGCAGCGTCA
FAE1-R	RT-qPCR	5'-GATGTTGCTTCGGAGCTTGA
FAD2-F	RT-qPCR	5'-ATCGCCGTCACCATTCCAAC
FAD2-R	RT-qPCR	5'-GGCAAGCGAACCCGTCATAC
FAD3-F	RT-qPCR	5'-CCATCGCTGCCGTGTATGTT
FAD3-R	RT-qPCR	5'-ATGGCCATGGTTCTGGTGGT
PDCT-F	RT-qPCR	5'-GACGGCGCGTGATATCGTCT
PDCT-R	RT-qPCR	5'-TTGCATCCCTACGAACACCGT
LPCAT1-F	RT-qPCR	5'-GCGCGGTTCAGATTCCACTT
LPCAT1-R	RT-qPCR	5'-AATAACCCGTGAGCCTGCGA
LPCAT2-F	RT-qPCR	5'-TGCGGTTCAGATTCCGCTTT
LPCAT2-R	RT-qPCR	5'-TGCTTGTTGCCACCGGTAAA
GPAT9-F	RT-qPCR	5'-GCATCCTGGTTGGGTTGGTC
GPAT9-R	RT-qPCR	5'-TGCAATTGGACAAACAGTGCAG
LPAT2-F	RT-qPCR	5'-GGCCGTCCCATAAAGTCCCT
LPAT2-R	RT-qPCR	5'-CTTGGCTGGGACGACTTTGG
WRI1-F	RT-qPCR	5'-CGCTAGGCATCACCACAACG
WRI1-R	RT-qPCR	5'-GCTTGGTTCACAGGGAACGG
GPDHc1-F	RT-qPCR	5'-GGTCAAATGCTGGCAAAGGG
GPDHc1-R	RT-qPCR	5'-TGCTTGCAGAATGGCCTGAG

Table S1. Primers used in this study.
The sizes of PCR products ranged from 80 to 300 nucleotides in length. F, forward primer; R, reverse primer.
